# Special Issue “Hepatobiliary and Pancreatic Cancers: Novel Strategies for of Diagnosis and Treatments”

**DOI:** 10.3390/jcm11133849

**Published:** 2022-07-02

**Authors:** Alessandro Coppola, Michele Fiore, Vincenzo La Vaccara, Tommaso Farolfi, Damiano Caputo, Sara Ramella

**Affiliations:** 1 General Surgery, Fondazione Policlinico Universitario Campus Bio-Medico, 00128 Rome, Italy; a.coppola@policlinicocampus.it (A.C.); d.caputo@policlinicocampus.it (D.C.); 2 Radiation Oncology, Campus Bio-Medico University, 00128 Rome, Italy; m.fiore@policlinicocampus.it (M.F.); s.ramella@policlinicocampus.it (S.R.); 3 General Surgery, Università Campus Bio-Medico di Roma, 00128 Rome, Italy; t.farolfi@unicampus.it

In recent years, hepato-pancreato-biliary (HPB) cancers have been increasing their incidence. In addition, they are also increasing their position in the list of the most deadly malignancies [1].

In fact, these groups of cancers have a very aggressive behavior with a very poor response rate to medical and surgical treatments.

However, despite this dramatic scenario, recently, several improvements in the field of oncology, radiotherapy and surgery were achieved, with a wide spread of new treatment protocols.

All these improvements derive from the concept that it is fundamental to utilize a multimodal approach with a team composed of surgeons, medical oncologists, radiation oncologists, radiologists, interventional radiologists, anesthesiologists and nutritionists [2].

Unfortunately, the great progress made in recent years slowed dramatically during the COVID-19 pandemic. In 70% of HPB surgery centers, waiting lists have been negatively impacted by the pandemic, often dictated by hospital healthcare management. Despite this, even taking into consideration the greater risks deriving from minimally invasive techniques, minimally invasive surgery maintains its role despite the COVID-19 pandemic, with the registered reduction in cases being proportional to the overall reduction in HPB surgical activity [3].

The current Special Issue, “Hepatobiliary and Pancreatic Cancers: Novel Strategies for Diagnosis and Treatments”, in the *Journal of Clinical Medicine*, is dedicated to collecting high-quality scientific contributions that mainly focus on modern approaches in diagnosis and treatments.

The first important remark to make is that there are huge differences in terms of therapeutic strategies and outcomes if we discuss liver or pancreatic cancers.

In addition, also looking inside a single organ, there are completely different approaches and outcomes.

Pancreatic adenocarcinoma remains one of the deadliest diseases, with a very poor survival rate, even after radical resection [4].

Due to that, several studies are reported or are ongoing focused on chemo and radio-chemotherapy regimens in an adjuvant, but mostly in a neoadjuvant, setting.

The Dutch Pancreatic Cancer Group recently published the long-term results of The PREOPANC Trial [5].

In total, 246 patients with resectable and borderline resectable pancreatic cancer were randomly assigned to neoadjuvant chemo-radiotherapy or upfront surgery. Despite the early results that showed no differences between the two approaches, the 5-year overall survival rate was 20.5% with neoadjuvant chemo-radiotherapy and 6.5% with upfront surgery.

A Korean phase II–III study evaluated gemcitabine-based chemo-radiation at a dose of 54 Gy, before or after surgery, followed by adjuvant chemotherapy with gemcitabine. This study was prematurely closed after the enrolment of 57 patients due to demonstrated superiority of preoperative treatment (OS 21 months vs. 12 months—R0 resection 51.8% vs. 26.1%) [6].

Encouraged by these results, there are several trials investigating the role of neoadjuvant regimens in the treatment of resectable pancreatic adenocarcinoma.

The Study Group of Preoperative Therapy for Pancreatic Cancer and Japanese Study Group of Adjuvant Therapy for Pancreatic Cancer recently published the study protocol of their randomized phase II/III trial, the Prep-02/JSAP05, aiming to demonstrate if neoadjuvant treatments could offer better prognosis in spite of upfront surgery [7].

We are currently waiting for the results of the reported trial and many other ongoing trials worldwide. In clinical practice, despite the classical anatomical definition of resectable, borderline and locally advanced pancreatic cancer, the concept of biological resectability is gaining momentum. This concept overcame the classical anatomical aspect and emphasizes the biological aggressiveness of the tumor in order to prepare patients for neoadjuvant treatment in spite of upfront surgery. In this setting, a neoadjuvant approach is justified by the possibility of increasing rates of radical intervention with negative resection margins.

To achieve that, biological neoplastic markers like Ca 19.9 play an important role.

In recent years, there have been many papers trying to identify the unexplored role of Ca 19.9 in patients with resectable pancreatic adenocarcinoma.

In 2021, our group published a retrospective work analysing the possible relation between high pre-operative levels of Ca19.9 and post-operative outcomes at the pathological report, also in relation to serum albumin levels.

In a group of 165 patients, a Ca19.9 > 37 U/mL in patients with normal albumin serum levels was strictly associated with nodal involvement at the histopathological evaluation, both in univariate and multivariate analysis.

Moreover, margin positivity after surgical resection was observed for Ca 19.9 at the cut-off >730 U/mL, calculated with an ROC curve analysis, despite low-positive and negative predictive values. On the contrary, no significant association was found with the need for vascular resection, even if a significant trend was shown for preoperative level of Ca19.9 > 78 U/mL [8].

Furthermore, the medical and socioeconomic impact of other pancreatic neoplasms, such as gastro-entero-pancreatic neuroendocrine tumours (GEP-NETs), is rapidly increasing.

The prognosis and impact of these cancers on the population is obviously different, but more favourable than in pancreatic adenocarcinoma. Nevertheless, the rising incidence and long follow-up period in the curative, as well as in the palliative, setting forces us to develop new strategies for early diagnosis and curative strategies for these tumours as well [9].

In addition, pancreatic surgery is burdened by high rates of postoperative complications, such as pancreatic fistula, biliary fistula, sepsis or haemorrhage.

Among these, the most important for both incidence and high mortality rates is the pancreatic fistula. As established by the International Study Group of Pancreatic Surgery (ISGPS), the fistula can be defined as a “biochemical leak” due to the lack of clinical importance.

In particular, grade B requires a change in the postoperative management and Grade C refers to those post-operative pancreatic fistulae that require reoperation or lead to single or multiple organ failure and/or mortality attributable to the pancreatic fistula [10].

Therefore, the early diagnosis of pancreatic fistula represents, to date, one of the greatest challenges for pancreatic surgeons. Many preoperative scores have been developed in order to identify those patients with higher risk to develop pancreatic fistula [11].

In the last few years, new strategies have been developed in order to prevent or improve the management of pancreatic fistula (PF).

Intraoperative study of the biliary microbacterial flora of patients undergoing pancreatoduodenectomy or total pancreatectomy was also investigated due to the relation between bacterobilia and postoperative morbidity, mostly due to infectious complications. Prevalence of polymicrobial biliary cultures with Escherichia coli, Klebsiella pneumoniæ, Enterococcus fæcalis and Enterococcus fæcium were significantly associated with post-operative PF; therefore, an antibiotic therapy tailored to the results of intraoperatively collected biliary samples may improve outcomes of pancreaticduodenectomy [12].

As well as pancreatic surgery, great progress has also been made in hepatobiliary surgery.

For example, the weapons available to fight liver metastasis from colorectal cancer are now multiple and increasingly advanced. Over the past 30 years, the overall survival in patients with liver metastases has increased from 20% at two years in the 1990s to more than 50% at five years today [13,14].

Key steps for this progressive increase in overall survival were the multidisciplinary approach, the great advances in chemotherapy and the parenchymal spearing hepatectomy, as well as an increasingly aggressive attitude towards metastasis that led surgeons to advanced procedures, such as ALPSS (Resection And Partial Liver Segment 2/3 Transplantation With Delayed Total Hepatectomy) until liver transplantation [15,16].

Unfortunately, till now, the same progress in overall survival have not been achieved in other liver cancers, such as cholangiocarcinoma or hepatocarcinoma.

Hepatocellular carcinoma (HCC) is the most common primary liver cancer with rates of incidence and mortality that have been rapidly increasing the in last few years. The five-year survival rates for HCC diagnosed at early stages is more than 70% compared with 5% when diagnosed at an advanced stage, often due to the impossibility of surgical resection, which is currently the only curative treatment [17].

Cirrhosis represents the main risk factor for HCC, with up to a 30% of patients developing HCC. Patients with cirrhosis have up to a 6% risk per year of developing HCC, so it is clear that regular surveillance may allow for early detection and an increased access to potentially curative therapies, such as liver resection or transplantation.

In the observational study published by Haq in 2021, the HCC surveillance was associated with earlier disease stage at presentation resulting in improved overall survival from diagnosis. More than the type of surveillance (Ultrasound Scan, AFP or both and their timing), the key factors were the adherence to the surveillance program that should be the key component of the initial discussion with the patient.

Controversy about the clinical and cost effectiveness of HCC surveillance will continue until large randomized controlled trial have been performed [18].

Hepatic resection is the treatment of choice for patients with HCC for non-cirrhotic liver. Where hepatic resection is not feasible, the treatment indications for HCC that appear in cirrhotic liver will be followed. Selection of cirrhotic patients who are candidates for local treatments cannot be based on rigid parameters, but on a comprehensive evaluation of the patient, including performance status, comorbidities, liver function, number and site of lesions and the extent of resection planned to achieve surgical radicality. The considerable complexity of this multiparametric assessment requires a multidisciplinary team with appropriate expertise.

Intrahepatic cholangiocarcinoma (ICC) represents the second most common primary malignancy of the liver with an increasing incidence. Unfortunately, only one-third of patients have access to liver resective surgery or, more recently, liver transplants, which are the only strategies with curative intent.

Therefore, identifying patients eligible for surgery becomes essential. Next to elevated levels of Ca-19.9, a poorly differentiated tumour or microvascular invasion that represents well-known prognostic factors for the survival of these patients, also preoperative identification of malnutrition (that afflict until 50% of patients with ICC), may be fundamental in patients undergoing liver resection and should not be performed using single clinical parameters, but performing a complete preoperative evaluation of the nutritional status [19].

As the Guest Editor team, we would like to thank all of the contributing authors for their valuable input. Ultimately, we sincerely appreciate and thank the reviewers for their insightful remarks and the JCM team’s support.
